# Process Analysis of Anaerobic Fermentation Exposure to Metal Mixtures

**DOI:** 10.3390/ijerph16142458

**Published:** 2019-07-10

**Authors:** Yonglan Tian, Huayong Zhang, Lei Zheng, Shusen Li, He Hao, Meixiao Yin, Yudong Cao, Hai Huang

**Affiliations:** Research Center for Engineering Ecology and Nonlinear Science, North China Electric Power University, Beijing 102206, China

**Keywords:** heavy metals, biogas production, substrate biodegradation, enzyme activity, microbial communities, methanogens

## Abstract

Anaerobic fermentation is a cost-effective biowaste disposal approach. During fermentation, microorganisms require a trace amount of metals for optimal growth and performance. This study investigated the effects of metal mixtures on biogas properties, process stability, substrate degradation, enzyme activity, and microbial communities during anaerobic fermentation. The addition of iron (Fe), nickel (Ni), and zinc (Zn) into a copper (Cu)-stressed fermentation system resulted in higher cumulative biogas yields, ammonia nitrogen (NH_4_^+^-N) concentrations and coenzyme F_420_ activities. Ni and Zn addition enhanced process stability and acetate utilization. The addition of these metals also improved and brought forward the peak daily biogas yields as well as increased CH_4_ content to 88.94 and 86.58%, respectively. Adding Zn into the Cu-stressed system improved the abundance of *Defluviitoga*, *Fibrobacter* and *Methanothermobacter*, the degradation of cellulose, and the transformation of CO_2_ to CH_4_. The bacterial and archaeal communities were responsible for the degradation of lignocelluloses and CH_4_ production during the fermentation process. This study supports the reutilization of heavy metal-contaminated biowaste and provides references for further research on heavy metals impacted anaerobic fermentation.

## 1. Introduction

The rapid increase in population, urbanization and industrialization has given rise to the excessive discharge of contaminants into the environment [1]. Unlike organic contaminants, heavy metals are toxic, not biodegradable and can be accumulated in living organisms [1]. Thus, reliable, environmentally-friendly technologies such as phytoremediation are required to treat wastewaters [2,3]. Phytoremediation involves the removal of pollutants by rapidly growing aquatic plants via plant uptake and metabolism. Unfortunately, phytoremediation produces a large amount of biomass which might be a potential source of secondary pollution [4,5]. Anaerobic fermentation is a relatively more efficient process for biomass waste reduction [4,5]. During anaerobic fermentation, microorganisms require trace amounts of different metals for their optimal growth and performance [6]. In general, heavy metals promote biogas production at certain concentrations [5,7,8,9] but inhibit anaerobic fermentation at higher concentrations [8,10,11]. However, the co-occurrence of different metals may induce complex reactions which may hinder the efficient utilization of biowaste.

Since Cu, Fe, Ni and Zn are widely distributed in terrestrial and aquatic systems, plants contaminated with those metals are often used in anaerobic fermentation [8,12,13,14]. The impacts of Cu, Fe, Ni and Zn, separately, on anaerobic fermentation were studied in recent decades. Table 1 summaries the previous studies about the impact of Cu, Fe, Ni and Zn on biogas production. Previous studies in the past decade have shown that, individually, these metals often enhance biogas production at certain concentrations [7,8,9,15]. However, most of the studies were focused on the general performances, i.e., biogas yields in the presence of single or mixed metals.

Early studies found <10–160, 700–2800, 65–180 and 50–630 μg/g Cu, Fe, Ni and Zn, respectively in methanogens (including 10 species of *Methanosarcina*, *Methanococcus*, *Methanobacterium*, and *Methanobrevibacter*, etc.) [16]. After investigation of the uptake and mass balance of trace metals for CH_4_ producing bacteria, Zhang et al. suggest that the contents of Cu, Fe, Ni and Zn in fermentation liquid should be greatly increased to get a high productivity of CH_4_ fermentation [17]. However, at relatively high concentrations, Cu inhibits substrate degradation and microbial growth as it alters the physiological steady state of the fermentation process [18,19]. In the methanogenic upflow anaerobic sludge blanket (UASB) granule, Cu has been shown to be more toxic than chromium (Cr), cadmium (Cd), Zn, Ni and lead (Pb) to volatile fatty acid (VFA)-degrading organisms [11].

Since almost every metalloenzyme involved in the pathway of biogas production contains multiple Fe_2_S_2_, Fe_3_S_4_, or Fe_4_S_4_ clusters, Fe is essential for biogas production [20,21,22]. The addition of Fe often stimulates biogas production by extending the gas production peak and enhancing cellulase activities [7]. The addition of 1500 mg/L Cu Fe-based bimetallic nanoparticles (nZVI/Cu) increased biogas production three times the control (no nZVI/Cu added) [23].

Most prosthetic groups in bacterial and archaeal enzymes contain Ni [24]. For example, CO dehydrogenase (CODH), a key enzyme in conversion of acetate to biogas, contains a Ni/Fe-S and a corrinoid/Fe-S component [25,26]. The enzymatic reduction of methyl coenzyme M to CH_4_ during methanogenesis by Methyl-coenzyme M reductase (MCR), an enzyme exclusively found in methanogenic archaea [27], involves a Ni containing cofactor called F_430_ [21,26,28]. Hence, Ni addition has been shown to increase the cumulative biogas yields by improving the efficiency of the first peak stage as well as bringing forward the second peak stage [9]. Ni was previously shown to act synergistically in Ni–Cu, Ni–Mo–Co and Ni–Hg systems but antagonistically in Ni–Cd and Ni–Zn systems (as reviewed by [29]). In contrast, Zn addition has been shown to be beneficial to enzymes required for acetoclastic and hydrogenotrophic CH_4_ production [15].

Considering the ubiquity of metal contamination, understanding the impact of metal mixtures on anaerobic fermentation is imperative in assessing their effect on waste minimization and biogas production. However, mechanistic investigations on the effects of combinations of Cu, Fe, Ni and Zn on the anaerobic fermentation process remain scarce. Therefore, the purpose of this study was to assess the impact of Fe, Ni and Zn on the anaerobic fermentation of Cu-containing waste biomass (i.e., mixed corn stover and cow dung). We evaluated the effect of metal mixtures on biogas properties, process stability, substrate degradation, enzyme activity as well as the microorganisms, in particularly methanogens during the fermentation process.

## 2. Materials and Methods

### 2.1. Experimental Materials

Corn stover collected from the farmland in Tongzhou, Beijing, China, in November 2016, was used as the feedstock. The corn stover was harvested by cutting 10 cm above the ground, then diced into pieces 5–10 cm in length, and then air dried until moisture levels were below 10%. After drying, the stover was ground into powder and passed through a 10-mesh sieve. Fresh cow dung collected from the Yanqing base, Beijing Dairy Cattle Centre, was mixed with stover as part of the feedstock and as the microbial inoculum. Prior to feeding to the reactor, the fresh cow dung was stored at 4.0 °C. No additional inoculum was used to initiate the fermentation. Table 2 shows the characteristics of the corn stover and the cow dung. Section 2.3 provides a description of the methods used to determine these physicochemical characteristics.

### 2.2. Anaerobic Fermentation Experiment

Experiments were conducted in anaerobic fermenters (30 L total volume and 20 L available volume, YGF 300/30, Shanghai Yangge Biological Engineering Equipment Co., Ltd., Shanghai, China) for 28 days at 55 ± 1.0 °C (auto controlled) as previously reported [5]. Prior to each experiment, the fermenters were cleaned and autoclaved. The digester contents were thoroughly stirred using a three-layer stirrer introduced in the middle of each fermenter for 30 min prior to sampling and measuring physicochemical parameters.

The feedstocks were comprised of a mixture of corn stover (0.8 kg dry weight) and cow dung (0.8 kg dry weight). The total solids (TSs) of the substrate in the fermenters was adjusted to 8% by adding distilled water. At the beginning of fermentation, 10.0 mg Cu/L (0.536 g of CuCl_2_·2H_2_O) was added into four fermenters. Approximately 10.0 mg Fe/L, 2.0 mg Ni/L, and 2.0 mg Zn/L were added to three of the fermenters by adding 0.712 g FeCl_2_·4H_2_O, 0.162 g NiCl_2_·6H_2_O and 0.083 g ZnCl_2_, respectively. There were four treatments with 10.0 mg/L Cu (Cu treatment), 10.0 mg/L Cu with 10.0 mg/L Fe (Cu + Fe treatment), 10.0 mg/L Cu with 2.0 mg/L Ni (Cu + Ni treatment) and 10.0 mg/L Cu with 2.0 mg/L Zn (Cu + Zn treatment). These concentrations of the metals were suggested to be stimulatory for biogas production according to the previous studies [7,8,9] and pre-experiments. Finally, the fermenters were purged with N_2_ gas for 5 min to remove oxygen.

### 2.3. Chemical and Microbial Analyses

Biogas yields and pH values were automatically measured at 09:00 h every day [8]. Solid, liquid and gas samples were collected every three days at 09:00. TSs were measured by weighing the samples after drying at 105 °C for 24 h. Volatile solids (VSs) were measured after treating the samples in a muffle furnace at 550 °C for 1 h. Total nitrogen (TN) was measured by the indophenol blue colorimetric method after being digested by concentrated sulfuric acid and 30% hydrogen peroxide [37]. Total organic carbon (TOC) was measured by potassium dichromate volumetric method [37]. Cellulose, hemicellulose and lignin in solid were determined by cellulose, hemicellulose and lignin Enzyme-Linked Immunosorbent Assays kit (ELISA, Qingdao Kebiao Testing and Research Institute Co. LTD, Qingdao, China), respectively. Chemical oxygen demand (COD) in the supernatant was obtained by the potassium dichromate method after sample centrifugation at 5000 rpm for 10 min. Ammonia nitrogen (NH_4_^+^-N) was measured by Nessler’s reagent method [38]. The cellulase and coenzyme F_420_ activities in the supernatant were determined according to the standard method after centrifugation at 4000 rpm for 5 min [39]. The coenzyme M was measured by botany coenzyme M ELISA kit (Qingdao Kebiao Testing and Research Institute co. LTD). Samples for VFA analysis were passed through a 0.45-μm nitrocellulose membrane filter and frozen prior to analysis. The concentrations of VFAs were measured using a gas chromatograph (GC-2014, Shimadzu Co., Kyoto, Japan) with a flame ionization detector (FID). Standards of C2–C5 fatty acids (chromatographically pure) were used for quantification. The VFA concentration was expressed as mg/L of individual species (C2–C5 fatty acids). CH_4_ contents in biogas were measured by a gas chromatograph (GC-2014C, Shimadzu Co., Japan) equipped with a GDX–401 column with H_2_ as the carrier gas. Detection was performed with a thermal conductivity detector (TCD).

The measurement of microbial sequences were conducted by the Novogene Co. Ltd. (Beijing, China) after centrifuging the samples at 8000 rpm and 4 °C for 3 min. Briefly, the genomic DNA of the samples on the 7th, 13th and 19th day were extracted by the Cetyltrimethylammonium Ammonium Bromide (CTAB) method [40]. After the extraction, the samples were diluted to a concentration of 1 ng/L with sterile water. Then the diluted genomic DNA was used as the template for PCR amplification. PCR amplification of the V3–V4 hypervariable region of bacterial 16S rDNA was performed using universal primers 338F (5′-ACTCCTACGGGAGGCAGCAG-3′) and 806R (5′-GGACTACHVGGGTWTCTAAT-3′) [41]. The archaeal primers used to amplify the V3–V4 hypervariable region of archaeal 16S rDNA were 344F (5′-ACGGGGYGCAGCAGGCGCGA-3′) and 806R (5′-GGACTACVSGGGTATCTAAT-3′). All primers included Illumina barcode sequences for multiplexing each sample. The library construction was conducted with TruSeq^®^ DNA PCR-Free Sample Preparation Kit. After the Qubit and Q-PCR quantification, the constructed library was qualified and HiSeq2500 PE250 was used for sequencing. The obtained sequencing data were stored in Fastq files.

### 2.4. Data Analysis

After removing the barcode and primers’ sequences from the sequencing data (FASTQ files), the reads were matched with fast length adjustment of short reads software (FLASH, V1.2.7, http://www.cbcb.umd.edu/software/flash) for raw Tags and then sieved for clean Tags. Clean Tags were cut out and the lengths filtered by referencing the quantitative insights into microbial ecology (QIIME V1.9.1, http://qiime.org/scripts/split_libraries_fastq.html) quality control process. The obtained Tags were treated by removing the chimeric sequence through comparison with the detection chimeric sequence (Gold database) yielding the final effective Tags as the targets. The cluster analysis of effective Tags was conducted using Uparse software (V7.0.1001, http://drive5.com/uparse/). The operational taxonomic units (OTUs) were clustered with identity >97%. Species annotation of the OTUs representative sequence was carried out using Mothur method and SSUrRNA database (define threshold of 0.8–1.0). The microbial communities were then obtained after annotation.

The data in the study were the average of triplicate treatments. Error bars represent the standard errors of the mean: SEM = SD/$\sqrt{n}$, where SD is the standard deviation. One-way analysis of variance (One-Way ANOVA) and Pearson correlation analysis were performed in the Statistical Package for the Social Science (SPSS, 17.0, Chicago, IL, USA) software at 0.05 and 0.01 levels of significance represented by * (*p* < 0.05) and ** (*p* < 0.01), respectively.

## 3. Results and Discussion

### 3.1. Biogas Properties of Cu-Treated Fermenters Combining with Fe, Ni and Zn

#### 3.1.1. Biogas Yields

**Figure 1:**
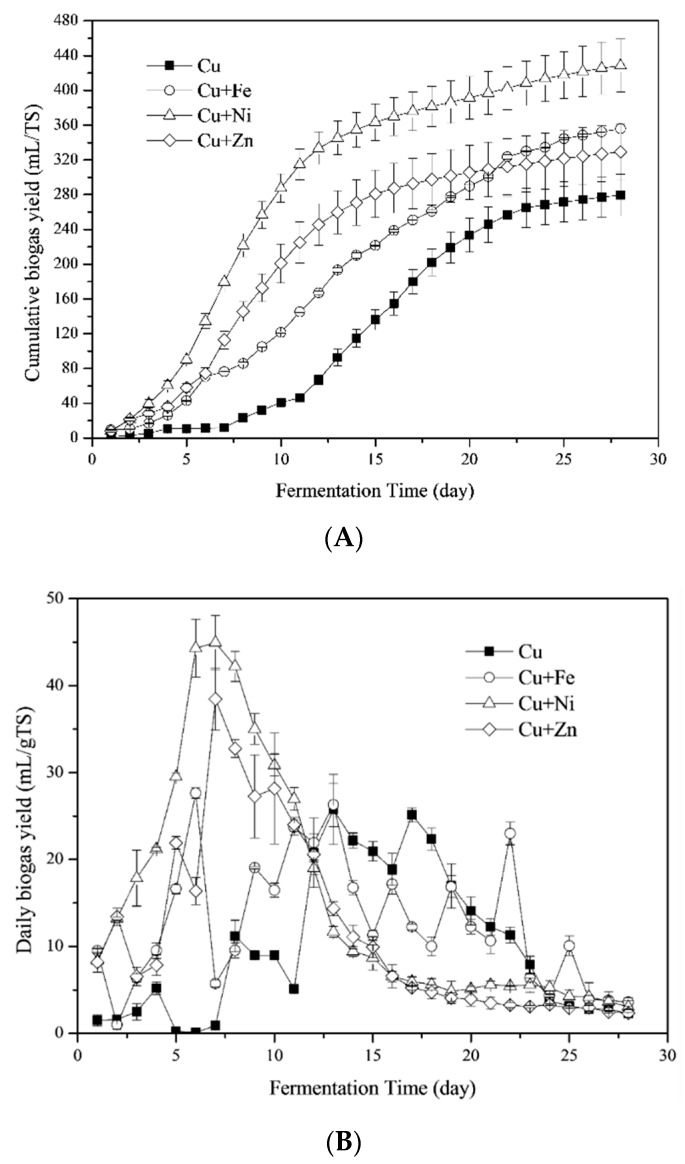

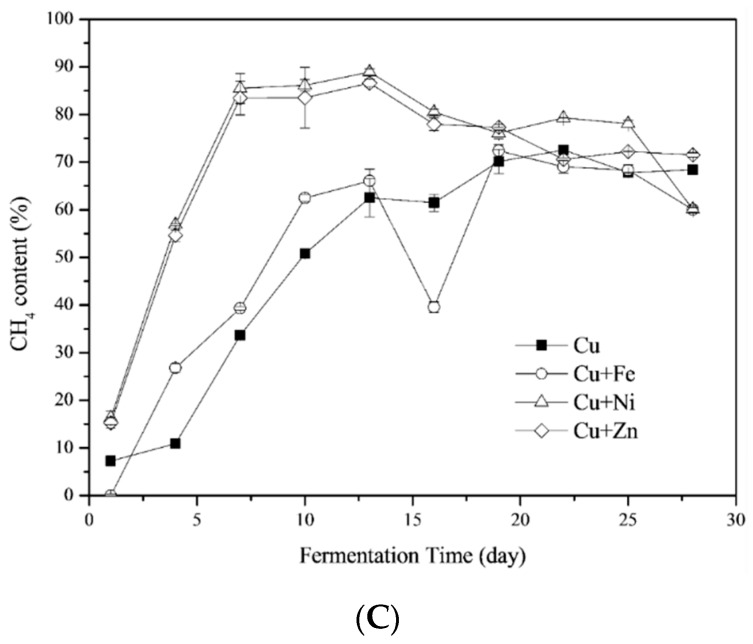
Cumulative biogas yields (**A**); daily biogas yields (**B**); and CH_4_ contents (**C**) during the fermentation process with combination of Cu and Fe, Ni and Zn.

Figure 1a shows the impact of Fe, Ni and Zn combined with Cu addition on cumulative biogas yields. For the Cu treatment, cumulative biogas yields were 279.46 ± 2.29 mL/g TS, which was much higher than the yields of anaerobic fermentation with *Phragmites* straw and cow dung as feedstock under 37.0 ± 1.0 °C [8]. The Cu + Ni treatment produced more biogas than other treatments while the Cu treatment obtained the lowest biogas yields. The cumulative biogas yields of Cu + Ni, Cu + Fe and Cu + Zn treatments were 53.33, 27.27 and 17.80%, respectively, which were higher than the Cu treatment. Thus, combining Fe, Ni and Zn with Cu promoted biogas production.

The stimulatory effect of combining metals on daily biogas yields were high during the initial 12 days of the fermentation period (Figure 1b). The highest daily biogas yields were 25.76, 27.63, 44.95 and 38.45 mL/g TS for Cu, Cu + Fe, Cu + Ni and Cu + Zn treatments, respectively. Distinctively high daily biogas peaks for Cu + Ni and Cu + Zn treatments on the 7th day were observed. The Cu + Fe and Cu treatments showed significant variations during the fermentation process. Despite yielding the lowest biogas, after the 11th day, the Cu treatment had a sharp increase in cumulative biogas yields as shown in Figure 1a. These results show that the addition of Fe, Ni and Zn into the Cu-containing anaerobic fermentation system could enhance the yield and quicken the daily biogas peak. Furthermore, these results explained the different growth process of cumulative biogas yields in the metal mixtures treatments. According to the previous studies, the growth process of cumulative biogas yields was generally expressed as a diauxie growth for those fermentation process with straw and cow dung as feedstocks when there was no metal added or one kind of metal added [2,5,7,8,9]. In the present study, the growth process in the Cu treatment was diauxie, while that in the Cu + Fe, Cu + Ni and Cu + Zn treatments was one-phase decomposition since the second biogas peak was brought forward and occurred together with the first one.

#### 3.1.2. CH_4_ Yields

The highest CH_4_ contents were 72.57, 72.41, 88.94 and 86.58% for Cu, Cu + Fe, Cu + Ni and Cu + Zn treatments, respectively. On the 1st day of fermentation, CH_4_ contents were low in the Cu and Cu + Fe treatments (Figure 1c). In contrast, the Cu + Ni and Cu + Zn treatments had a higher CH_4_ content, suggesting Ni and Zn probably accelerated the initiation of the fermentation process. The CH_4_ contents of the Cu- treatments gradually increased, equaling Cu + Fe treatment after 13 days and then Cu + Zn after 22 days. The CH_4_ contents in Cu + Ni and Cu + Zn treatments were similar from the 1st day to the 23rd day, marked by a rapid increase in the first 7 days and then plateauing until the 13th day, followed by a gradual decrease. CH_4_ contents in the Cu treatment were similar with the previous study, in which 30 and 100 mg/L Cu addition resulted in a CH_4_ content of 86.72 and 77.35%, respectively [8]. Moreover, CH_4_ contents in Cu + Fe and Cu + Ni groups were higher than that in Fe (67.90%) and Ni (70.41%) experiments [7,9]. Therefore, the addition of Cu improved CH_4_ production and further addition of Ni and Zn enhanced the stimulatory effect.

### 3.2. Process Stability as Indicated by the Variation of pH Values

**Figure 2:**
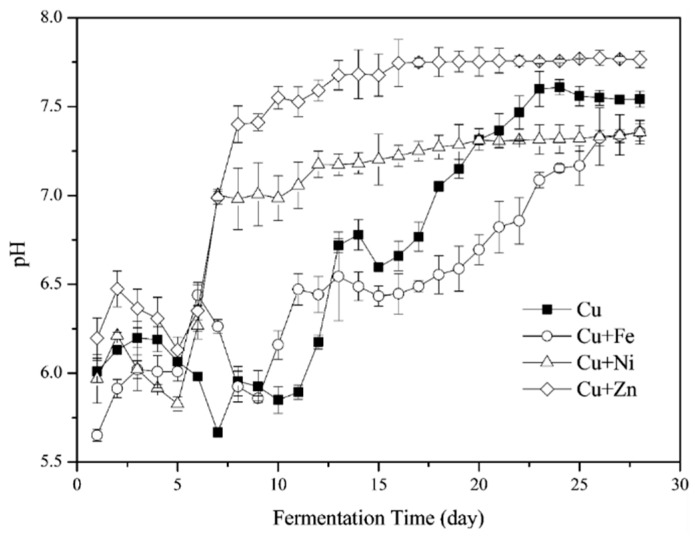
The pH values following the addition of Cu together with Fe, Ni, or Zn during the fermentation process.

pH values increased slightly after the fermentation started (Figure 2). There was considerable fluctuation in pH values from the Cu- and Cu + Fe treatments. The pH increased remarkably after the 11th and 10th day, respectively. After the two-day surge, pH values in these two treatments decreased slightly and then increased again until it stabilized at a pH of 7.54 and 7.35 in the Cu and Cu + Fe treatments, respectively. A similar trend was reported in previous research [8]. For Cu + Ni and Cu + Zn treatments, pH values decreased after the 1st day and recovered after the 5th day. The increase in pH values in these two groups decreased after the 7th and 8th day with the final pH values plateauing at around 7.35 and 7.77, respectively.

Taking the whole process into account, pH values for all the four treatments were generally located in the optimal pH ranges for the function of most methanogenic bacteria [42]. Moreover, Fe addition did not change the pH variation while Ni and Zn addition benefited the process stability. However, in some studies the pH values decreased during the initiation of the fermentation process probably due to accumulation of the acidic components hydrolyzed from organic materials [5,8]. In this study, the feedstocks were efficiently used for methanogenesis with no accumulation of acidic components.

### 3.3. Substrate Biodegradation

#### 3.3.1. Variations of Ammonia Nitrogen (NH_4_^+^-N) Concentrations

**Figure 3:**
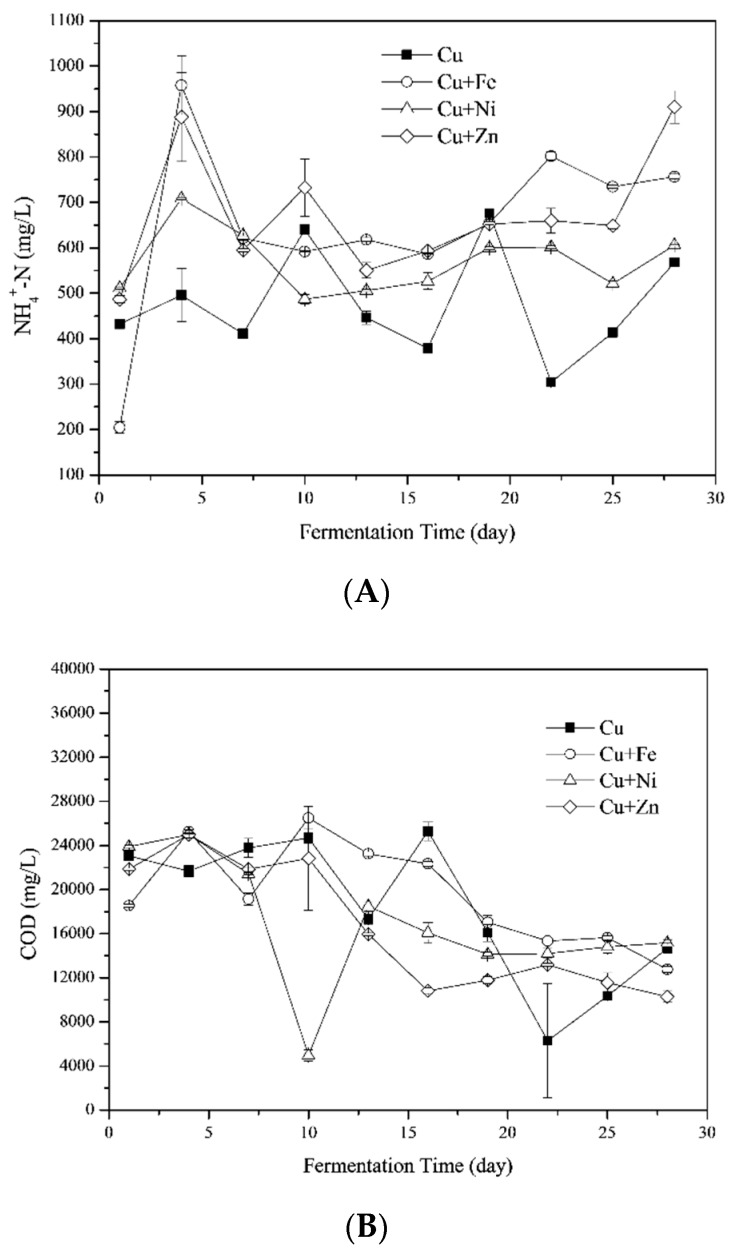
Ammonia nitrogen (NH_4_^+^-N) (**A**) and chemical oxygen demands (CODs) (**B**) during the fermentation process with Fe, Ni and Zn addition combined with Cu.

Total ammonia, consisting of ammonium ions and free ammonia, is produced during the anaerobic degradation of proteins, urea, and nucleic acids [43]. It is a basic nutrient for microorganism growth at NH_4_^+^-N concentrations below 200 mg/L [44]. However, it has been shown that NH_4_^+^-N concentrations ranging from 0.6 to 14 g/L inhibited the methanogenic activity (reported by [45] and reviewed by [29]) depending on different experimental conditions [46]. The effects of combining metals on NH_4_^+^-N concentrations during the fermentation process are shown in Figure 3a. The average NH_4_^+^-N concentrations were 476.63 ± 37.36, 652.83 ± 61.88, 569.31 ± 23.62 and 671.82 ± 43.40 mg/L for Cu, Cu + Fe, Cu + Ni and Cu + Zn treatments, respectively. Thus, NH_4_^+^-N concentrations in the present study were not likely to inhibit biogas production.

Compared to the previous study, in the presence of Cu, the NH_4_^+^-N concentration in this study was higher than that with *Phragmites* straw and cow dung as feedstocks [8]. On one hand, the nitrogen content in the cow dung was higher in this study (Table 2). On the other hand, the *Phragmites* straw in the previous study was pretreated by acid, which reduced the pH values after the experiment started up and after the 15th day. Moreover, the NH_4_^+^-N concentration was reported to be positively correlated to pH values in the presence of Cu [47]. Therefore, the acidic environment as indicated by the reduced pH values in the previous study resulted in lower NH_4_^+^-N concentrations than the present study. Furthermore, the addition of Fe, Ni and Zn benefited for the degradation of substrate containing nitrogen which contributed to the higher NH_4_^+^-N concentrations.

#### 3.3.2. Chemical Oxygen Demands (COD)

The soluble organic components in the fermenter, shown as COD, originated from the hydrolysis of liquefied large molecules (i.e., long chain natural polymers of the substrate) by extracellular enzymes [22,48]. The impacts of combining metals addition on COD are shown in Figure 3b. In Cu treatment, COD was generally stable during the first 10 days of fermentation, which was different from the previous study [8], since the organic components in the liquor was efficiently used for CH_4_ production as proved by Figure 1b. The COD was then decreased in the later stage of fermentation, which indicated that organic components in the liquid phase were utilized for biogas production. COD of Cu + Fe and Cu + Zn treatments showed the same trend, while COD of Cu + Ni treatment decreased sharply on the 10th day corresponding with the high VFA concentrations on the same day.

The average COD during the entire fermentation process was 18,323.99 ± 2055.90, 19,578.36 ± 1448.09, 16,816.34 ± 1834.62, and 16,508.78 ± 1821.49 mg/L for the Cu, Cu + Fe, Cu + Ni and Cu + Zn treatments, respectively. The COD at the beginning of fermentation in Cu treatment was much higher than the previous study [8]. The possible explanation was the differences in feedstocks and fermentation temperatures. For the present study, the VS contents of the cow dung was higher than the previous study which indicated more organic matters available for microorganisms. Moreover, thermophilic fermentation generally induced higher metabolic rates than mesophilic fermentation. That is, the fermentation temperature of 55.0 ± 1.0 °C in this study resulted in more organic matters in the fermenter liquor than the previous study (37.0 ± 1.0 °C) [8]. There was no significant difference between the treatments. Hence, the VFAs and other distinct components might be more sensitive to the impact of metal mixtures on the different stages of fermentation.

#### 3.3.3. Responses of Volatile Fatty Acids (VFAs)

**Figure 4:**
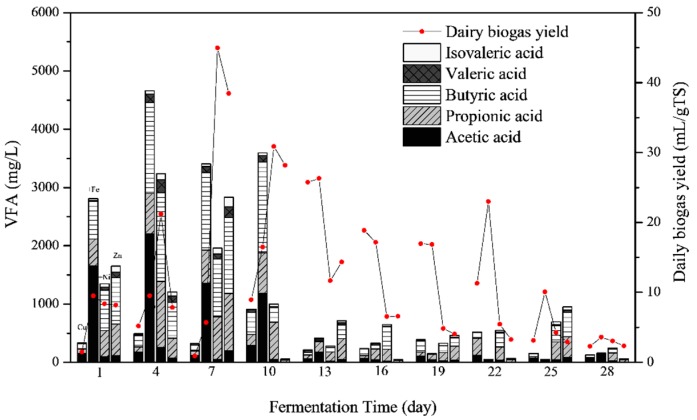
Volatile fatty acids (VFAs) concentrations in the liquid phase during the fermentation process. For each day, the column from left to right shows the VFA compositions of Cu, Cu + Fe, Cu + Ni and Cu + Zn treatments, respectively. The compositions of VFA were shown by stacked bars in the order of acetic acid (black), propionic acid (bias), butyric acid (transverse) and valeric acid (grid) from bottom.

The total VFA concentrations and their compositions, i.e., acetic acid, propionic acid, butyric acid and valeric acid, are shown in Figure 4. As reported in a previous study [8], the total VFA concentrations were generally high at the early stage of fermentation and subsequently declined as the fermentation process progressed. However, the total VFA concentrations were generally lower than in the previous research [8]. This result was coincidedwith the higher biogas yields in this study, indicating the efficient utilization of VFAs for biogas production.

During the first 7 days, total VFA concentrations (which corresponded with higher biogas yields) of Cu + Fe, Cu + Ni and Cu + Zn treatments were higher than the Cu treatment (Figure 1b). This suggested that VFAs were more efficiently produced and consumed for biogas production in these treatments. During this period, the pH values remained high, since alkaline components such as NH_4_^+^-N were being generated. On the 10th day, an increase in the VFAs concentration and daily biogas yields in Cu and Cu + Fe treatments was observed. In contrast, the limitation of VFAs in Cu + Ni and Cu + Zn treatments resulted in a decrease in the daily biogas yields (Figure 1b).

The main compositions of VFAs varied with metal addition. During the first 7 days, acetic acid was the main component, followed by butyric acid > propionic acid > valeric acid in the Cu and Cu + Fe treatments. However, the order of the VFA components by amount was butyric acid >propionic acid > acetic acid > valeric acid in Cu + Ni and Cu + Zn treatments. Acetic acid was efficiently used during methanogenesis; hence the higher biogas and CH_4_ yields in Cu + Ni and Cu + Zn treatments. After the 10th day, the concentrations of all VFA compositions in Cu + Ni and Cu + Zn treatments decreased. Moreover, the low efficiency of transferring C3–C5 VFAs into acetic acid limited biogas production. However, higher acetic acid was recorded in the Cu and Cu + Fe treatments which probably contributed to the higher daily biogas yields during the late stage of fermentation.

#### 3.3.4. Degradation of Lignocelluloses

Lignocelluloses are mainly composed of cellulose, hemicellulose, lignin [49]. As shown in Table 3, lignin and cellulose contents were comparable and higher than the hemicellulose contents in all the treatments. When metal mixtures were added into the fermentation reactors, the degradation of lignocelluloses differed significantly. The addition of Fe and Zn into Cu-containing fermenters enhanced the degradation of lignin and cellulose significantly, which resulted in a decrease in total lignocelluloses contents (One-Way ANOVA, *p* < 0.01). In contrast, Ni addition did not improve the degradability of the feedstocks.

**Figure 5:**
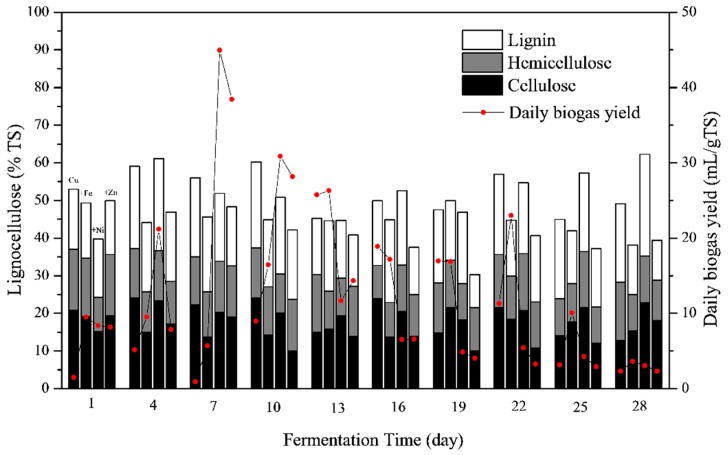
Lignocelluloses contents during the fermentation process. For each day, the column from left to right shows the lignocelluloses contents of Cu, Cu + Fe, Cu + Ni and Cu + Zn treatments, respectively. The compositions of lignocelluloses were shown by stacked bars in the order of cellulose (black), hemicellulose (gray) and lignin (blank) from bottom.

Figure 5 shows the effects of metal mixtures on lignocelluloses compositions during the fermentation process. On the 1st day of fermentation, the addition of Fe, Ni and Zn into the Cu-containing fermenter promoted the degradation of lignocelluloses, in particular cellulose and hemicellulose, resulting in higher daily biogas yields than the Cu treatment. Afterwards, the lignocelluloses contents of Cu + Ni treatment were generally higher than Cu + Fe and Cu + Zn treatments, especially for the cellulose contents. Therefore, Ni probably did not contribute significantly to the cellulose degradation. The high biogas yields of the Cu + Ni treatment was likely due to the degradation of hemicellulose and other non-lignocellulose components during the early stage of fermentation. A previous study on the anaerobic fermentation of *Phragmites* straw and cow dung found Cu addition enhanced the degradation of lignin and hemicellulose [8]. In the present study, the addition of Zn increased the degradation of cellulose. Therefore, Zn combining Cu was suggested for the further degradation of lignocellulose and higher biogas yields.

### 3.4. Responses of Enzyme Activity

#### 3.4.1. Cellulase

**Figure 6:**
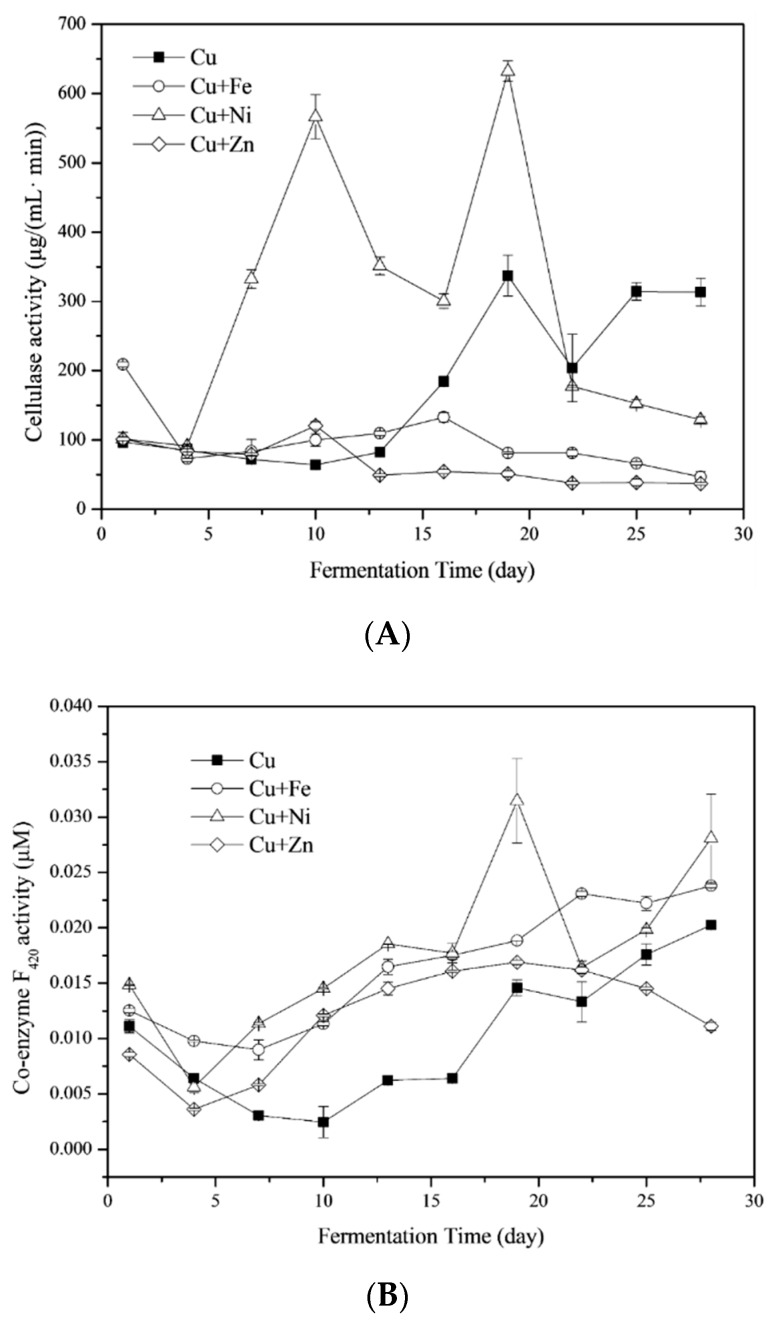

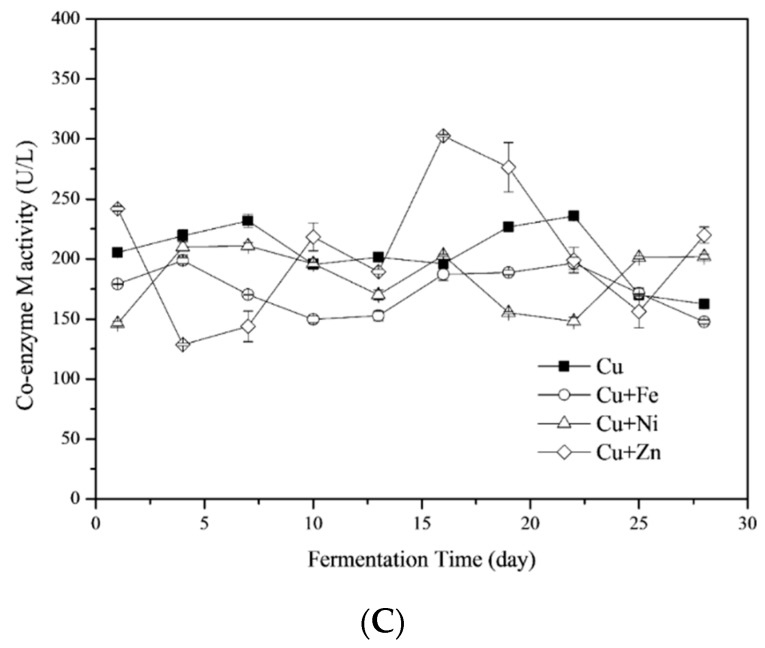
Responses of cellulase (**A**), coenzyme F_420_ (**B**) and coenzyme M (**C**) to Fe, Ni and Zn combined with Cu.

Cellulase activity is often influenced by cellulose contents and COD in the liquid phase of the substrate. There was a 13-day lag in the cellulase activity of the Cu treatment (Figure 6a), which corresponded with the cellulose content and daily biogas yields (Figure 1b). The trend of cellulase activity in the Cu treatment was different from a previous study on the anaerobic fermentation of *Phragmites* straw and cow dung [8]. However, the subsequent degradation of cellulose suggests cellulase activity was probably influenced by the type of feedstocks.

The average cellulase activities were 175.43 ± 35.18, 98.45 ± 14.42, 283.5 ± 60.65 and 65.36 ± 9.26 μg/(mL min) for Cu, Cu + Fe, Cu + Ni and Cu + Zn treatments, respectively. The addition of Ni induced significantly higher cellulase activities than Cu alone (One-Way ANOVA, *p* < 0.05). However, high cellulase activity did not result in a corresponding significant reduction in cellulose contents probably because Ni addition enhanced the degradation of hemicellulose and other non-lignocellulose components, resulting in the production of cellulose in the total solid (TS) (Table 3). On the contrary, the cellulase activities in the Cu + Zn treatment were significantly lower than in Cu treatment (One-Way ANOVA, *p* < 0.05) while the cellulose contents were significantly reduced. The results suggested lower cellulase activity may not be causally related to lower cellulose degradation. Hence, there is a need for a comprehensive study on the mechanism of cellulose degradation in the anaerobic fermentation of biowaste impacted by metals.

#### 3.4.2. Coenzyme F_420_

The activity of coenzyme F_420_ is an index—a measure of methanogens activity [50,51]. Similar to the cellulase activities, the coenzyme F_420_ activities in the Cu treatment declined at the beginning of fermentation and recovered after the 13th day (Figure 6b). The same trend was found in Cu + Fe and Cu + Ni treatments. Differently, coenzyme F_420_ activities in the Cu + Zn treatment decreased at the later stage of fermentation (after the 19th day). The average coenzyme F_420_ activities were 0.010 ± 0.002, 0.016 ± 0.002, 0.018 ± 0.002 and 0.012 ± 0.001 μM for Cu, Cu + Fe, Cu + Ni and Cu + Zn treatments, respectively. Fe and Ni addition significantly improved the activity of coenzyme F_420_ (One-Way ANOVA, *p* < 0.05 for Cu + Fe treatment and *p* < 0.01 for Cu + Ni treatment).

Coenzyme F_420_ plays a critical function in the H_2_/CO_2_ pathway for CH_4_ production [8]. In most systems, approximately 70% of the carbon fixed in CH_4_ was derived from acetate. Only minor amounts, up to approximately 30%, were deduced from the H_2_/CO_2_ pathway [52,53]. However, the relative contribution of H_2_/CO_2_ versus acetate as metabolic precursors for methanogens can be quite different in different anaerobic environments [53]. As shown in Figure 6b and Figure 1c, the coenzyme F_420_ activities of all four treatments declined while CH_4_ contents increased after the experiment started. During this period, the acetate pathway likely played a main role in CH_4_ production. Afterwards, the increase in coenzyme F_420_ activities contributed to the transformation of H_2_ and CO_2_ and the rapid increase in CH_4_ contents.

#### 3.4.3. Coenzyme M

Though methanogens may differ in terms of their preference for initial substrates (formate, acetate and/or H_2_/CO_2_), all of them synthesize a common compound, coenzyme M (2-mercaptoethane sulphonate) [54]. The limitation of the coenzyme M availability to methanogens subsequently suppressed the activity of MCR in methanogens, which in turn reduced the methanogenesis rate [55]. The activities of coenzyme M fluctuated during the fermentation process and the variation in the Cu + Zn treatment was more dramatic than other treatments (Figure 6c). The average activities of coenzyme M were 204.39 ± 7.85, 174.16 ± 6.05, 184.24 ± 8.35 and 207.63 ± 17.81 U/L for Cu, Cu + Fe, Cu + Ni and Cu + Zn treatments, respectively. There was no significant difference between different treatments (One-Way ANOVA, *p* > 0.05).

Zn took part in the functioning of enzymes involved in methanogenesis such as coenzyme M methyltransferase [16]. Ni in methanogens was expected to influence coenzyme M concentrations [56], which was however not found in the present study, possibly due to the low concentrations and availability of Ni. Furthermore, the increment of coenzyme M contents in the cells of *Methanosarcina acetivorans* was triggered by Cd^2+^ stress [57]. The responses of coenzyme M to metal stress needed to be studied in detail together with the metal analysis in the cells of methanogens.

### 3.5. Impacts of Metal Mixtures on the Microbial Communities

#### 3.5.1. Structure of Microbial Communities

**Figure 7:**
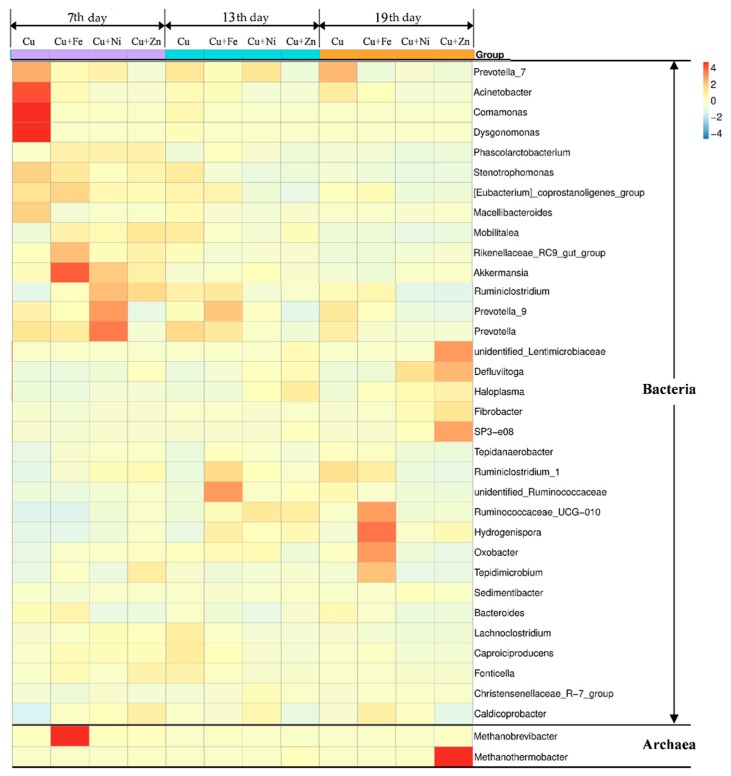
Microbial communities annotated on the level of genus in Cu, Cu + Fe, Cu + Ni and Cu + Zn treatments on the 7th, 13th and 19th day of fermentation. Colors represent the normalized sequences number of the operational taxonomic units (OTUs) in the samples (blue: low sequences number of OTUs; red: high sequences number of OTUs).

Microbial community variations annotated at the level of genus are shown in Figure 7. In the 35 most distributed microorganisms on the level of genus, there were only two archaea with others being bacteria. The metal mixtures exerted different influences on both bacterial and archaeal communities as the fermentation progressed. For the bacterial communities, *Prevotella*_7, *Acinetobacter*, *Comamonas* and *Dysgonomonas* were the dominant microorganisms of the Cu treatment on the 7th day. *Prevotella*, previously classified in the genus *Bacteroides*, is an obligate anaerobic gram-negative rod-shape bacterium [58]. It has a limited ability to ferment amino acid and previous studies found that it negatively correlated with a number of pathways involved in amino acid metabolism and carbohydrate metabolism, including short-chain fatty acid metabolism as well as pathways involving nitrogen, sulfur, and CH_4_ metabolism [59]. Although *Acinetobacter* and *Comamonas* require oxygen for survival, they were found in the Cu treatment and further research on their role in anaerobic fermentation might be necessary.

In the Cu + Fe treatment, the dominant bacteria were *Rikenellaceae*_RC9_gut_group and *Akkermansia*. To date, all cultured members of the family *Rikenellaceae* were described as anaerobic, mesophilic, rod-shaped bacteria that usually ferment carbohydrates or proteins [60]. Therefore, the detected microbial communities in the Cu + Fe treatment supported the carbohydrates and proteins degradation. In the Cu + Ni treatment, the main bacteria were *Ruminclostridium*, *Prevotella*_9 and *Prevotella*. As previously reported, *Ruminiclostridium cellulolyticum* produce extracellular multi-enzymatic complexes called cellulosomes, which efficiently degrade the crystalline cellulose and the cell wall [61]. Thus, *Ruminiclostridium* in the Cu + Ni treatment contributed to the decrease in cellulose on the 7th day (comparing with the cellulose contents on the 4th day in Figure 5). The Cu + Zn treatment had a lower sequence number of the OTUs than other treatments, with the *Ruminclostridium* slightly higher than other bacteria.

On the 13th day, the sequence number of unidentified_*Ruminococcaceae* in the Cu + Fe treatment increased while other bacteria decreased. *Ruminococcaceae* belonged to the *Firmicutes* which have been shown to be capable of assimilating polysaccharides such as cellulose and starch [62,63]. The bacteria in all other groups were detected with lower abundance.

On the 19th day, the *Prevotella*_7 in the Cu treatment recovered and were the dominant genus (Figure 7). For Cu + Fe treatment, the sequence number of *Ruminococcaceae* _UCG_010, *Hydrogenispora*, *Oxobacter* and *Tepidimicrobium* were enhanced. Members of the genus *Tepidimicrobium* are anaerobic, moderately thermophilic and neutrophilic, with the temperature range for optimum growth 25–67 °C and the pH range for optimum growth 5.5–9.5 [64]. *Tepidimicrobium* spp. grew organotrophically on a number of proteinaceous substrates, amino acids, and carbohydrates [64] and produced acetic acid, ethanol, H_2_ and CO_2_. *Oxobacter*, an endospore-forming microorganism, obligately anaerobic, was able to catabolize pyruvateto into acetate and CO_2_ [65]. Meanwhile, the coenzyme F_420_ activity in the Cu + Fe treatment was high on the 19th day (Figure 7). Therefore, the H_2_ and CO_2_ were efficiently generated and used for CH_4_ production at this time.

For the Cu + Zn treatment, the sequence number of unidentified_*Lentimicrobiaceae* were highest followed by a SP3-e08, *Defluviitoga* and *Fibrobacter*. A new isolate L3 of *Defluviitoga tunisiensis* was able to degrade cellulose since genes encoding non-cellulosomal cellulases have been previously identified in the islaltes’ genome [66]. Acetate, H_2_ and CO_2_ were the probable end products of the fermentation process. *Fibrobacter succinogenes*, a predominant cellulolytic rumen bacterium, was previously suggested to be capable of degrading cellulose via a very efficient cellulolytic system [67]. The high abundance of *Defluviitoga* and *Fibrobacter* in the Cu + Zn treatment probably enhanced the decrease in cellulose obseved in Figure 6.

For the archaeal communities, on the 7th day, *Methanobrevibacter* in the Cu + Fe treatment was dominant. All the *Methanobrevibacter* species are hydrogenotrophs [68] and use H_2_ and/or formate as substrate for their CH_4_ production [69]. The positive correlation between *Methanobrevibacter* and CH_4_ metabolism was previously reported [59]. Thus, *Methanobrevibacter* in the Cu + Fe group contributed to the generation of CH_4_ from H_2_. On the 19th day, the sequence number of *Methanothermobacter* was highest in Cu + Zn treatment. *Methanothermobacter* were strictly anaerobic with the fastest growth observed between 55 and 65 °C [70]. Their energy metabolism is followed by the reduction of CO_2_ to CH_4_, with H_2_ as an electron donor but some cells can use formate as electron donor instead [70]. It indicated that Zn addition into the Cu-containing group enhanced the growth of *Methanothermobacter* which promoted the transformation of H_2_/CO_2_ to CH_4_.

#### 3.5.2. Methanogens and Their Relationships with Fermentation Parameters

**Figure 8:**
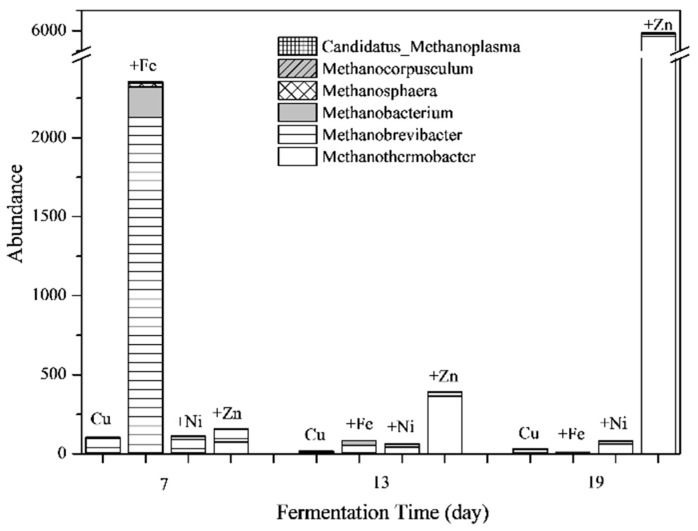
Responses of the absolute abundance of methanogens to Fe, Ni and Zn combined with Cu addition.

The absolute abundance of the methanogens at different stages of fermentation are shown in Figure 8. It was found that the total abundance of methanogens in the Cu treatment was lowest during the whole fermentation process. Ni addition increased the total abundance of methanogens slightly. For the Cu + Fe treatment, the total abundance of methanogens on the 7th day were much higher than other treatments with *Methanobrevibacter* as the dominant genus, followed by *Methanobacterium*. These result agreed with a previous study that found Fe was required by enzymes from *Methanobacterium* [54] as well as detected Ni in *Methanobrevibacter* [71]. However, the *Methanobrevibacter* was not dominant in Cu+Ni treatment probably due to the low concentrations and bioavailability of Ni. For Cu + Zn treatment, the total abundances of methanogens were higher than other treatments on the 13th and 19th day. In particular, the main methanogens in this treatment on the 19th day were identified as *Methanothermobacter*, which were >700 times higher than the Cu treatment.

**Figure 9:**
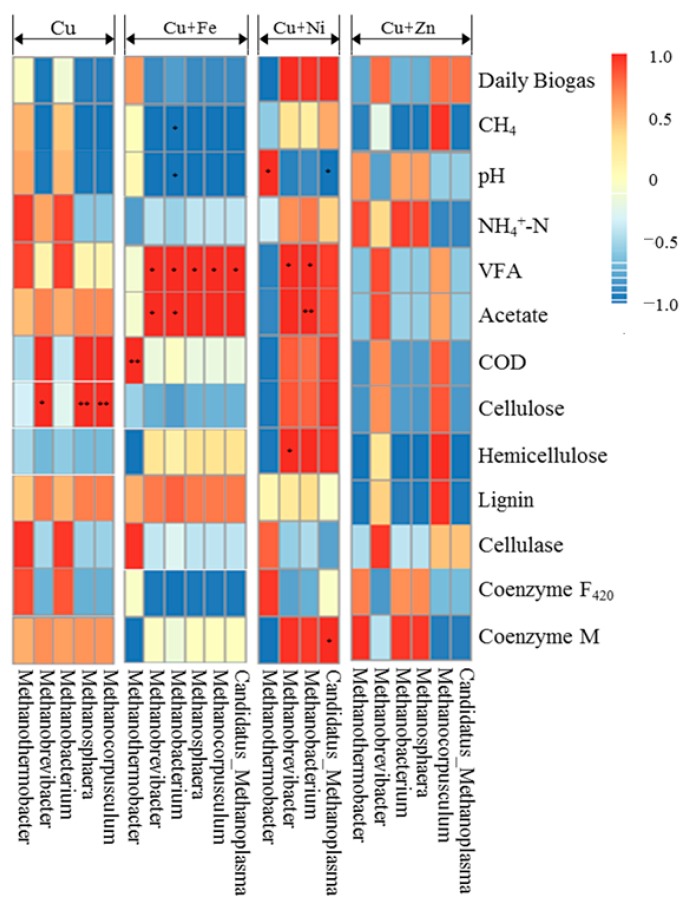
Pearson correlations between methanogens and fermentation parameters (*, *p* < 0.05; **, *p* < 0.01). NH_4_^+^-N, ammonia nitrogen; COD, chemical oxygen demands; VFA, volatile fatty acids. Different colors represent the correlation coefficients from −1 (blue) to 1 (red) as shown in the right.

The relationships between the methanogens and fermentation parameters were determined using Pearson correlation analysis and the results are shown in Figure 9. For Cu treatment, *Methanobrevibacter* (*p* < 0.05), *Methanosphaera* (*p* < 0.01) and *Methanocorpusculum* (*p* < 0.01) were positively correlated to the cellulose contents. However, cellulose contents were not correlated to methanogens in the compounding metal treatments. For Cu + Fe treatment, *Methanobacterium* was negatively correlated to CH_4_ contents and pH values (*p* < 0.05). *Methanothermobacter* was positively correlated to COD (*p* < 0.01). *Methanobacterium* and *Methanobrevibacter* were positively correlated to total VFA and acetate concentrations (*p* < 0.05). *Candidatus_Methanoplasma*, *Methanocorpusculum*, and *Methanosphaera* were positively correlated to total VFA concentrations (*p* < 0.05). For Cu + Ni treatment, *Methanothermobacter* was positively correlated to pH values (*p* < 0.05), which was contrary to *Candidatus_Methanoplasma* (negatively, *p* < 0.05). *Methanobrevibacter* and *Methanobacterium* were found to be positively correlated to total VFA *(p* < 0.05) and the former was positively correlated to hemicellulose contents (*p* < 0.05), while the later was positively correlated to acetate concentrations (*p* < 0.01). *Candidatus_Methanoplasma* was positively correlated to the coenzyme M activities (*p* < 0.05) while no other detected methanogens were correlated to cellulase, coenzyme F_420_ or coenzyme M in the Cu or other metal mixtures treatments. There were no significant relationships between the methanogens and fermentation parameters in the Cu + Zn treatment.

According to the abovementioned results, it was found that although the addition of metal mixtures induced the variation of methanogens during the fermentation process, this variation could not adequately explain the impacts of metals on biogas production. It seems the archaeal communities together with bacteria communities determined the degradation of feedstocks as well as the generation of CH_4_.

## 4. Conclusions

This study investigated the effects of a combination of Cu and Fe, Ni and Zn on the anaerobic fermentation of corn stover mixed with cow dung as feedstocks. The addition of metal mixtures, particularly Ni and Zn, improved the biogas yields and resulted in the one-phase decomposition process. The stimulatory mechanism of metal mixtures was demonstrated by improvements in process stability, efficiency in transformation and utilization of VFAs, and higher activities of coenzyme F_420_, and better degradation of lignin and cellulose. The effect of metal mixtures was strongly associated to the microbial communities rather than the methanogens themselves during the fermentation process. The addition of Fe increased the absolute abundance of *Methanobrevibacter* at the early stage of fermentation while the addition of Zn increased the absolute abundance of *Methanothermobacter* at the later stage of fermentation, which contributed to the generation of CH_4_ from CO_2_/H_2_. The results of this study manifested that metal contained biowastes were of large potential in producing biogas. The control and regulation of metal mixtures in the fermenter is important in improving fermentation efficiency. Future research and practice should focus on developing a mixed microbial agent for improving degradability and CH_4_ yields.

## Figures and Tables

**Table 1:** Previous studies about the impact of Cu, Fe, Ni and Zn on anaerobic fermentation.

Feedstocks	Metals	Reactor Type and Volume	Temp (°C)	HRT (d)	Biogas Yield	CH_4_ Yield	References
*Phragmites *straw + Cow dung	Cu	Pilot (30 L)	37 ± 1	33	110.59 mL/gTS	Maximum 86.72%	[8]
*Phytolacca americana* L.	Cu	Batch (0.7 L)	37	50	0.12 L/gVS	52%	[30]
*Zea mays* L.	Cu	Batch (0.7 L)	37	50	0.45 L/gVS	~70%	[30]
*Brassica napus* L.	Cu	Batch (0.7 L)	37	50	0.40 L/gVS	~70%	[30]
*Elsholtzia splendens*	Cu	Batch (0.7 L)	37	50	0.27 L/gVS	56%	[30]
*Oenothera biennis* L.	Cu	Batch (0.7 L)	37	50	0.39 L/gVS	56%	[30]
*Phragmites *straw + Cow dung	Fe	Batch (0.25 L)	35 ± 1	26	26.20–32.46 mL/gTS	Maximum 67.9%	[7]
Synthetic waste	Fe	UASB (9 L)	35	9	0.362 L/g COD removed	80.5–93.0%	[20]
*Lemnaceae* + Poultry manure	Fe	Batch (1 L)	32 ± 2	50–80	0.281 L/g VS	65–80%	[31]
*Lemnaceae* + Poultry manure	Fe	Semi-cont. (25 L)	32 ± 2	8.3–16.6	22.76 L/d	NR	[31]
Synthetic model substrate for maize silage	Ni	Batch (1 L)	35	30	NR	188–404 L_N_/kg organic dry matter	[32]
Model substrate for maize	Ni	Semi-cont. (5 L)	35	~60	0.6–8.0 L_N_/d	36–55%	[33]
*Phragmites *straw + Cow dung	Ni	Batch (0.25 L)	35 ± 1	26	27.49–32.70 mL/gTS	Maximum 70.41%	[9]
*Azolla pinnata* R.Br	Fe, Cu, Cd, Ni, Pb, Zn, Mn and Co	Batch (2 L)	37	36–42	132–189 L/kg	45–83%	[10]
*Lemna minor* L.	Fe, Cu, Cd, Ni, Pb, Zn, Mn and Co	Batch (2 L)	37	36–42	132–176 L/kg	43–85%	[10]
Seaweed	Cd, Cu, Ni, Zn	Batch (0.5 L)	37	30	NR	0.09–0.12 L_N_ CH_4_/g VSa (44.4–49.7%)	[34]
Seaweed	Cd, Cu, Ni, Zn	UASB (0.8 L)	37 ± 1	8.8–0.5	0.22–3.04 L_N_ CH_4_/L·d	0.16–0.23 L_N_ CH_4_/g CODa (62.9–73.7%)	[34]
*Triticale*	Al, Ni, Zn, Co, U, La	Stirring reactor (5 L)	38 ± 1	NR	~780 L_N_/kgVS	440 L_N_/kgVS	[35]
*Brassica juncea*	Al, Ni, Zn, Co, U, La	Stirring reactor (5 L)	38 ± 1	NR	~640 L_N_/kgVS	425 L_N_/kgVS	[35]
*Helianthus annuus*	Al, Ni, Zn, Co, U, La	Stirring reactor (5 L)	38 ± 1	NR	~360 L_N_/kgVS	163 L_N_/kgVS	[35]
*Eichhornia crassipes*	Cu- and Cr-rich brass and electroplating industry effluent	Batch (NR)	35 ± 1	20	11.10–27.80 L/kg dw	29.80–63.82%	[36]
*Trapa bispinnosa*	Cu- and Cr-rich brass and electroplating industry effluent	Batch (>0.15 L)	35 ± 1	20	10.45–20.90 L/kg dw	27.00–57.04%	[36]

NR—not reported; HRT—hydraulic retention time; Semi-cont, semi-continuous reactors; UASB—upflow anaerobic sludge blanket; TS—total solid; VS—volatile solid.

**Table 2:** Characteristics of corn stover and the cow dung.

Characteristics	Corn Stover	Fresh Cow Dung
TS (%dry weight)	95.59 ± 0.23	16.49 ± 0.16
VS (% TS)	90.72 ± 0.24	84.00 ± 0.48
TN (% TS)	1.21 ± 0.03	3.22 ± 0.11
TOC (% TS)	13.94 ± 0.64	14.81 ± 0.37
Ratio of C/N	11.52 ± 0.05	4.45 ± 0.30
Cellulose (% TS)	20.19 ± 1.24	23.56 ± 1.47
Hemicellulose (% TS)	14.05 ± 2.25	16.41 ± 0.48
Lignin (% TS)	13.55 ± 0.07	15.41 ± 1.11
Cu (μg/g)	8.57 ± 0.20	38.63 ± 0.30
Ni (μg/g)	1.71 ± 0.37	1.78 ± 0.10
Cd (μg/g)	Negligible	Negligible
Zn (μg/g)	14.89 ± 1.61	152.44 ± 2.06
Fe (μg/g)	520.80 ± 67.03	610.80 ± 12.87
Co (μg/g)	0.34 ± 0.24	0.61 ± 0.03
Cr (μg/g)	8.33 ± 1.13	3.01 ± 0.63

Mean ± Standard Error. *n* = 3. TS—total solid; VS—volatile solid; TN—total nitrogen; TOC—total organic carbon.

**Table 3:** The average contents of cellulose, hemicellulose, lignin and total lignocellulose during the anaerobic co-fermentation of corn stover and cow dung in the presence of different metal mixtures.

Metals	Lignin (% TS)	Hemicellulose (% TS)	Cellulose (% TS)	Total Lignocellulose (% TS)
Cu	19.63 ± 0.85	13.23 ± 0.75	19.34 ± 1.46	52.21 ± 3.06
Cu + Fe	16.92 ± 0.90	11.44 ± 0.61	16.46 ± 0.83	44.83 ± 2.34 **
Cu + Ni	19.95 ± 1.15	12.05 ± 0.69	20.21 ± 0.74	52.21 ± 2.58
Cu + Zn	14.56 ± 1.03 **	12.34 ± 0.61	14.43 ± 1.18 **	41.34 ± 2.82 **

Mean ± Standard Error; *n* = 10; One-Way ANOVA; ** *p* < 0.01; TS—total solid.
